# Characterizing cellular membrane partitioning of DMSO using low-temperature Raman spectroscopy

**DOI:** 10.3389/fmolb.2023.1144059

**Published:** 2023-02-22

**Authors:** Troy Louwagie, Madeline Wagner, Rui Li, Guanglin Yu, Ashley Petersen, Allison Hubel

**Affiliations:** ^1^ Department of Mechanical Engineering, University of Minnesota Twin Cities, Minneapolis, MN, United States; ^2^ Department of Biomedical Engineering, University of Minnesota Twin Cities, Minneapolis, MN, United States; ^3^ Division of Biostatistics, University of Minnesota Twin Cities, Minneapolis, MN, United States

**Keywords:** cryopreservation, Raman spectroscopy, biotransport, cryoprotectants, cell membrane

## Abstract

Additives that help cells survive the stresses of freezing and thawing are known as cryoprotective agents (CPAs). Two different types of CPAs have been identified: penetrating and non-penetrating. Common penetrating CPAs include dimethylsulfoxide (DMSO) and glycerol. The location of a CPA (intracelluar or extracellular) is important for understanding the molecular mechanisms of action for the agent. Low-temperature Raman spectroscopy is a label-free method of detecting the location of CPAs at low temperature with high spatial resolution and chemical specificity. To this end, cells cryopreserved in DMSO using a variety of cooling rates and DMSO concentrations and imaged using Raman spectroscopy were analyzed using automated image analysis to determine the partitioning ratio (concentration of DMSO outside/concentration of DMSO inside the cell). The partitioning ratio was roughly 1 for Jurkat cells frozen at 1°C/min in varying concentrations of DMSO with the exception of 1% DMSO which had a partitioning ratio of 0.2. The partitioning ratio increased from 1 to 1.3 as the cooling rate increased from 1°C to 5°C/min. Different cell types, specifically sensory neurons cells and human induced pluripotent stem cells, exhibited differences in partitioning ratio when frozen in 10% DMSO and 1°C/min suggesting that differences in freezing response may result from differences in solute partitioning. The presence of intracellular ice changed the distribution of DMSO inside the cell and also the partitioning ratio.

## 1 Introduction

The storage of biological systems at sub-zero/cryogenic temperatures is most commonly called cryopreservation. This storage method is intended to inhibit degradation of the biological system. Cryopreservation is a critical element of the supply chain for a variety of applications including biomedical research, biotechnology, and cell therapy. Freezing cells enables manufacturing process development and safe transportation between collection site, manufacture, and use. Storing cells also enables safety and quality control testing during manufacturing.

Effective methods of cryopreservation require the use of specialized additives which help cells survive the stresses of freezing and thawing. These additives are commonly called cryoprotective agents (CPAs) (Murray and Gibson, 2022). Glycerol was the first CPA identified by Polge et al. (1949). Later, Lovelock and colleagues discovered the cryoprotective benefits of dimethylsulfoxide (DMSO) (Lovelock and Bishop, 1959). Since then, a wide variety of cryoprotective agents have been identified (see (Hubel, 2018) for review). There are two basic categories of CPAs: penetrating and non-penetrating. Large molecular weight CPAs such as polymers (e.g., hydroxyethylstarch), sugars (e.g., trehalose) and proteins (human serum albumin) do not penetrate the cell membrane unless the cell has specific transporters for that molecule. Small molecular weight additives such as DMSO, glycerol, and propylene glycol may penetrate the cell membrane (McGrath, 1998).

A variety of methods have been used to estimate the permeation of cryoprotective agents into cells. Most methods involve measurement of changes in cell volume with time when exposed to a solution with a given composition (McGrath, 1997) and then comparing the volumetric measurements to existing theoretical models for transport of water and cryoprotective agents (Kleinhans, 1998) in order to obtain estimates for the permeability of both cryoprotective agent and water. The permeability of water and solute is temperature dependent with permeability decreasing with decreasing temperature (McGrath and Diller, 1988). It has been postulated that transport across the cell membrane is negligible for temperatures < -35°C (Kleinhans and Mazur, 2009). The different measurement techniques have been largely indirect and none of them involve direct measurement of intracellular CPA concentration.

Raman spectroscopy has emerged as a powerful tool to investigate the freezing responses of various types of cells (Yu et al., 2021). This method is label-free and has high chemical specificity and spatial resolution. Low temperature Raman spectroscopy has been used to identify ice inside of cells (Yu et al., 2017), sites of damage during freezing and the location of cryoprotective agents (Yu et al., 2019). Raman spectroscopy is sensitive to the structure of hydrogen bond network, making it a suitable tool to examine the effects of cryoprotectants on the water hydrogen bond network at a low temperature. In this work, Raman spectroscopy and the resulting images will be analyzed to determine CPA concentration inside the cell compared to outside at low temperatures. The influence of formulated CPA concentration, cooling rate and cell type will be determined. The compartmentalization of CPA inside the cell as well as solute polarization outside the cell will be characterized. These studies will be important in understanding further the role of CPAs on the survival of cells during the freezing process. This investigation will focus on the distribution of dimethylsulfoxide (DMSO), a commonly used CPA. This approach, however, can be extended to other cryoprotective agents.

## 2 Materials and methods

### 2.1 Cell culture

A T-cell line (Jurkat cells, ATCC, TIB-1522) was incubated at 37°C in a 5% carbon dioxide atmosphere using high glucose RPMI-1640 (Life technologies, Carlsbad, CA) and 10% fetal bovine serum (qualified, Life technologies, Carlsbad, CA). Cells were grown in suspension and maintained at a concentration of (1–2) × 10^6^ cells/mL. The cells were washed twice in Dulbecco’s phosphate buffered saline prior to freezing studies using the temperature controlled stage (Yu et al., 2019).

The human induced pluripotent stem cells (hiPSCs) line DF-19-9-11 was used in this study (Li et al., 2018). These cells were cultured on Matrigel (hESC-qualified, LDEV-free; Corning) in Essential 8 medium (Thermo Fisher) at 37°C and 5% carbon dioxide atmosphere. The cells were passaged using ReLeSR (STEMCELL Technologies). hiPSC cultures were routinely tested for *mycoplasma* using the MycoAlert PLUS detection kit (Lonza). Freezing studies were performed with single cells after dissociation with Accutase (Innovative Cell Technologies) and gentle pipetting.

Using a commercially available kit, hiPSCs were differentiated into sensory neurons (Senso-DM, Anatomic Incorporated 7,007) and further matured (Senso-MM, Anatomic Incorporated 7,008). Briefly, hiPSCs were passaged as small cell aggregates using Versene at a split ratio of 1:20 onto Matrix 1. These cultures were cultured in the presence of Senso-DM formulations 24 h later, with subsequent formulations applied daily for 1 week in total. The differentiated cells were dissociated and replated onto a new substrate as per manufacturer’s instructions and allowed to mature in Senso-MM media for another 7 days with half media exchange every 2 days. Raman imaging of the cells were performed on Day 8 of the differentiation process before maturation.

For all cell types, cell pellets obtained using centrifugation were resuspended in 1%, 5%, and 10% DMSO in Normosol-R at a concentration of at least 25 million cells/mL, transferred into Corning tubes and incubated at room temperature for 30 min before Raman imaging. This concentration is used to ensure 2-4 cells are able to be imaged in the sample.

### 2.2 Temperature controlled stage

The temperature-controlled stage consisted of a four-stage Peltier (Thermonamic Electronics Corp. Jingxi, China) and a series 800 temperature controller (Alpha Omega Instruments Corp, Lincoln RI). Approximately 1–3 *μ*L of cell suspension was placed on the stage and covered with single layer mica (TED PELLA) to prevent evaporation/sublimation. Seeding temperature influences cell response. To eliminate variation due to seeding temperature, all samples were seeded at −4°C with a liquid nitrogen-cooled needle, purged with nitrogen gas to avoid condensation on top of mica, and then cooled at 0.5, 1, 5 or 10°C/min until −50°C and imaging studies were performed at that temperature.

### 2.3 Confocal Raman system

The WiTec Confocal Raman Microscope System Alpha 300R with UHTS300 spectrometer and DV401 CCD detector 600/mm grating were used to obtain confocal Raman spectroscopy samples. The WiTec spectrometer was calibrated with a mercury-argon lamp. A 532 nm wavelength Nd:YAG laser was used as the excitation source. A 100× air objective (NA 0.90; Nikon Instruments, Melville NY) was used for focusing the 532 nm excitation laser to the sample. Laser power at the objective was 10 mW as measured by an optical power meter (THORLABS, New Jersey). Resolution of the microscope was approximated as 0.3 *μ*m with Abbe’s diffraction formula for lateral resolution. Cells were imaged at the mid-plane of each cell.

### 2.4 Raman imaging and spectral analysis

Image spectral data was exported for each pixel from the WiTec Project 4.0 software. Area under isolated resonant peaks corresponds with the relative concentration of a given substance within the pixel for comparison across the pixels of that specific image. The peak wavenumber was chosen according for the substance of interest as shown in Table 1, and the width of the peak was determined using derivative thresholding. Each pixel in the image was examined for the peak of interest. The lower wavenumber of the peak of interest for that given pixel was chosen when the slope of the spectrum at relative wavelength x was 0.1 the slope of the spectrum at relative wavelength x+1 until the peak is found. A similar approach was used to determine the wavenumber associated with the upper limit of the peak. This sensitivity (0.1) was maintained amongst the different compounds of interest and resulted in a consistent width from image to image (between 4 and 8 steps of resolution, i.e., wavenumber 4.83 cm^−1^ per step). Intensity of various signal bands were computed using discrete trapezoidal area integration (MATLAB trapz()).

**TABLE 1:** Characteristic Raman spectral peaks of substances.

Substance	Wavenumber range (1/cm)	Initial wavenumnber (1/cm)	Assignment
Protein/Lipid (cell)	1,610–170	1,660	Amide I/alkyl C=C stretching
Ice	3,087–3,162	3,125	OH stretching
DMSO	650–740	673	Symmetric CS stretching
DNA	760–810	785	O-P-O Stretching of DNA

Background subtraction was completed by subtracting the minimum value in the peak to isolate the peak from background noise below. This process was completed for the following characteristic signals shown in Table 1. Intensities of each substance of interest were computed for all pixels. Images obtained using hiPSCs had 45 × 45 pixels with an integration time of 0.2 s per pixel and width of 12 *μ*m. Images obtained using Jurkat cells had 45 × 45, or 60 × 60 pixels with an integration time of 0.2 s per pixel and width of 12 or 15 *μ*m. A nearest neighbor average was used to eliminate the effect of cosmic rays (Cappel et al., 2010). A limited number of samples (5̃%) required multiple layers of ray removal where the nearest neighbor average was completed more than one time (limit 3). Cells with more than 10 pixels of scattered signal did not use cosmic ray removal and were not used for analysis.

### 2.5 Automated image analysis

**FIGURE 1:**
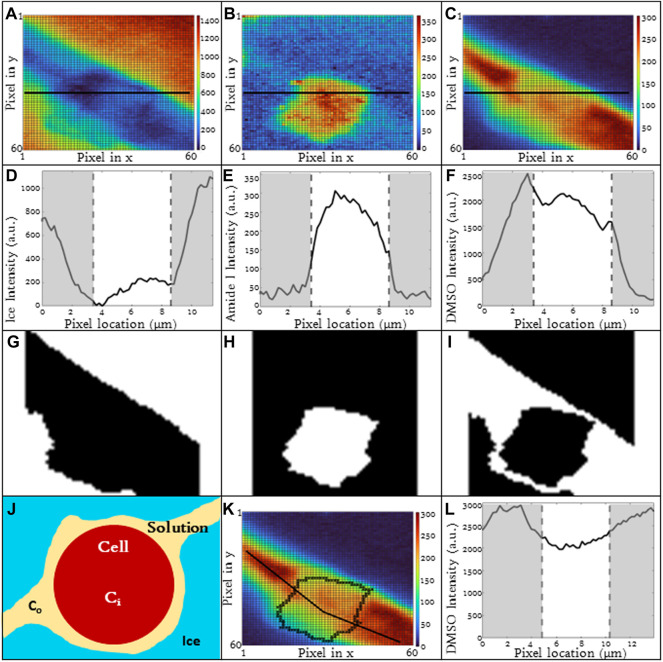
Raman heat maps of T-cell in 10% DMSO frozen using a -1°C/min cooling rate and imaged at a temperature of −50°C. Heat maps show intensity of **(A)** ice, **(B)** cell (amide I), and **(C)** DMSO. Intensity was defined as the area under the specified peak. This peak width was defined with derivative thresholding, and all background below the spectral trend was subtracted. Line plots across the center of each show variation of spectral peak intensity across the image for **(D)** ice, **(E)** cell, and **(F)** DMSO. Binary cell image from edge detection and cell morphology to deduce regions of interest in white. From left to right, white signifies **(G)** ice, **(H)** cell, and **(I)** unfrozen channel. **(J)** The partitioning ratio of CPA is defined as the ratio of the extracellular concentration divided by the intracellular concentration, a line plot across the cell demonstrates the variation in DMSO concentration across the cell and in the extracellular solution **(K,L)**. Pixels in **(A–C)** have a height and width of 200 nm. **(D**,**E**,**F,L)** regions of gray are in extracellular/ice regions and regions of white is intracellular.

Image spectra are analyzed to identify three different compartments: 1) cell, 2) ice and 3) extracellular solution. Cells that exhibit extensive intracellular ice formation are omitted from analysis using the automated image analysis system. Raman heat maps of a sample cell are given in Figure 1 for signals of interest (ice, Figure 1A, amide I, Figure 1B, DMSO, Figure 1C). One step of the automated cell analysis involved locating the cell boundary, which was performed using a series of operations in MATLAB image analysis software. Edge detection with a chosen sensitivity (0.5–0.9; most commonly 0.65) gave a preliminary estimate for the location of the cell edge. The next step involves applying a Sobel operator (discrete differentiation) to filter the edge (Cappel et al., 2010). Next, perpendicular line structuring elements are added to create a strel object for an initial binary outline of the cell. The lines are then dilated, and fill was added. The border is then cleared and smoothed to deliver the final cellular structure. The end result is a mask for each compartment. Exemplary masks for the extracellular solution, cell, and ice are given in Figures 1G–I. Line scans across the heat maps can also be used to identify the compartment (Figures 1D–F), and the cell boundary was identified in the same location with both masking and line scans. After determining the cell boundary, the partitioning ratio was determined. For the purpose of this investigation, the “partitioning ratio” (P) is defined as the average integrated peak value of DMSO in the extracellular channels, *C*_*o*_, divided by the average integrated peak value of DMSO inside the cell, *C*_*i*_ (Figure 1J).

$$P=\frac{C_{o}}{C_{i}}$$ (1)

DMSO line scans were analyzed (Figures 1K, L) and used to calculate partitioning ratio. This partitioning ratio compared to that obtained using the automated image analysis as described above. Ten Jurkat cells at 10% DMSO and 1°C/min were analyzed by visual inspection to validate partitioning ratio spatially. When selecting more than 50 pixels, the algorithm was within 5% of the hand calculation. Using line drawings through unfrozen space through the cell, the partitioning ratio was also within 5%, confirming the algorithm.

### 2.6 Jurkat cell freezing experiments

Post-thaw recovery of the Jurkat cells were determined at various DMSO concentrations. The cells were centrifuged for 10 min at 1,000 rpm and resuspended to a concentration of 6 million cells/mL 30 min before freezing. Preliminary studies demonstrated minimal cell losses (less than 5%) associated with this method of introduction and incubation. Cells were added in a 1:1 ratio with DMSO solutions at 2%, 10%, and 20% DMSO concentration to achieve a final concentration of 1%, 5%, and 10% DMSO. The addition of cryoprotection solutions occurred at room temperature and the cells were incubated for 30 min before freezing. A controlled-rate freezer (Kryo 560, Planer, Middlesex, UK) was used for freezing samples with a plunge (Steps 4–6) to induce nucleation around −4°C to reflect the same conditions performed under Raman spectroscopy. The freezing protocol used is given below:

Step 01: Start temp at 20°C.

Step 02: −10 °C/min to 0°C.

Step 03: Hold 15 min.

Step 04: −1°C/min to −4°C.

Step 05: −50 °C/min to −45°C.

Step 06: +15 °C/min to −12°C.

Step 07: −0.5 °C/min, −1°C/min, or −5°C/min depending on sample to −60°C.

Step 08: −10 °C/min to −100°C.

After the freezing procedure was completed, vials were stored in vapor phase for a minimum of 24 h before thawing.

### 2.7 Thawing and post-thaw assessment

Thawing was performed in a 37°C water bath and was completed under 3 min. Acridine orange (AO; Thermo Fisher) and propidium iodide (PI; Thermo Fisher) were used to stain cells for recovery calculations on a hemocytometer (Millipore Sigma). A 1:1 ratio of AO/PI and cells were used for counting on the hemocytometer.

### 2.8 Statistics

Independent replicates were used with sample size specified in the Results section per data set. Power analysis was performed to ensure sufficient sample size to achieve a power of 0.95. For statistical validation, two-sample t-tests with a 95% confidence level were performed. The null hypothesis was rejected when *p* < 0.05. Standard error was used for all error bars. Hand calculation without cellular thresholding was completed for several samples to validate partitioning ratio results.

## 3 Results

### 3.1 Spatial variations in composition

**FIGURE 2:**
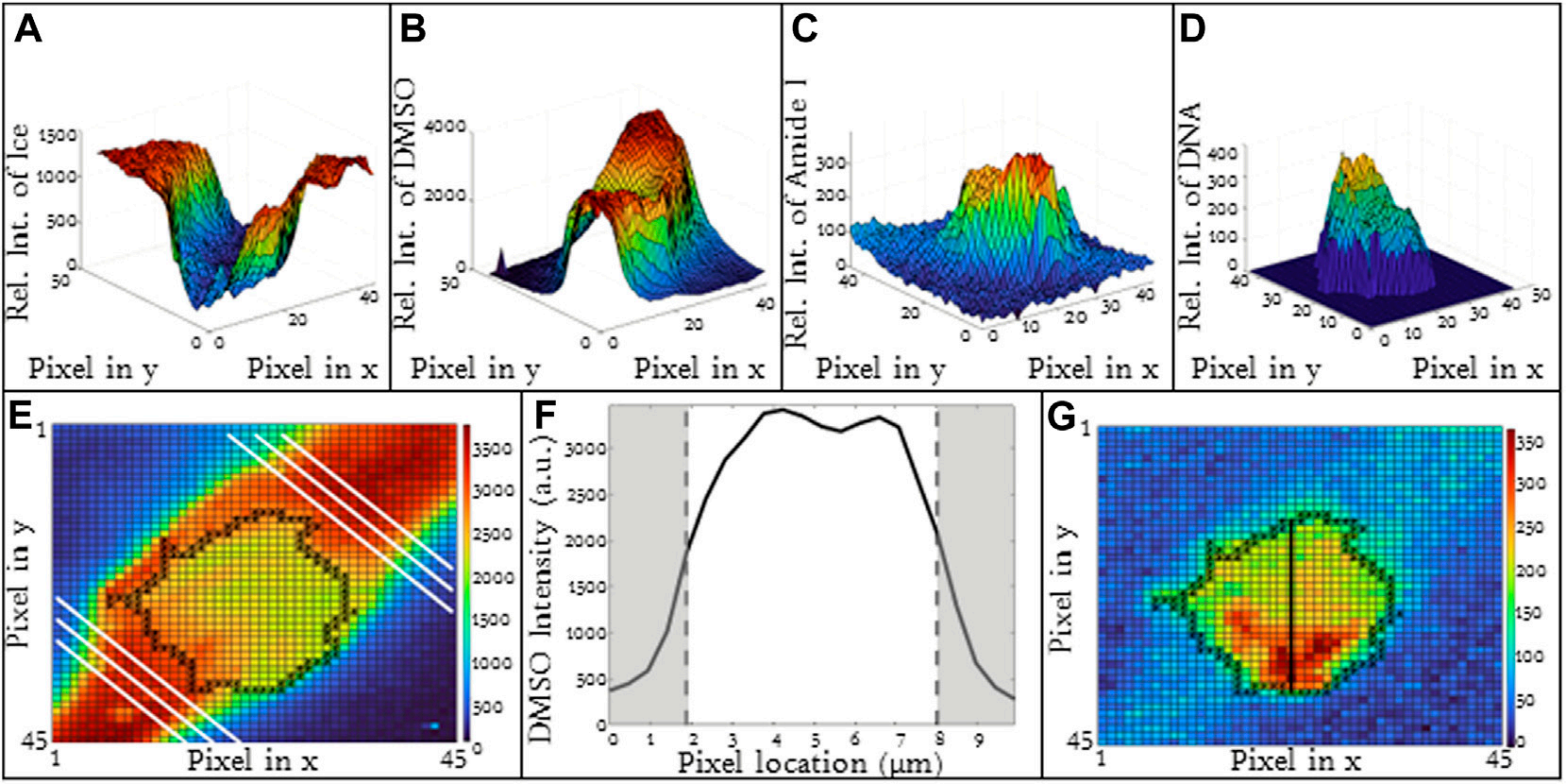
3D Raman heat maps for a T-cell frozen in a 10% DMSO solution using a cooling rate of −1°C/min and imaging at −50°C. Heat maps show intensity of **(A)** ice, **(B)** DMSO, **(C)** cell (amide I), **(D)** DNA. **(E)** Heat map of DMSO distribution in the extracellular space **(F)** average DMSO concentration in the extracelluar space between ice crystals indicated by the white lines as noted in 2E. **(G)** Raman heat map of DNA distribution in a Jurkat cell. Regions in red indicate high concentrations of DNA. Pixels in **(A)**, **(B)**, **(C)**, and **(G)** have a height and width of 333 nm. **(F)** Regions of gray indicate ice regions and white is extracellular space.

The spatial variation in ice, DMSO, Amide I and DNA is given in Figure 2 for a T-cell frozen in a 5% DMSO solution using a cooling rate of 1 °C/min. There are two compartments of interest: 1) intracellular and 2) extracellular. For the purpose of this investigation, the cell boundary separates the intracellular and extracellular spaces and the Amide I signal is used to denote the two compartments. In the extracellular space, ice is present outside the cell and the intensity of ice is high and uniform (Figure 2A). DMSO is found in the unfrozen solution surrounding the cell (Figures 2B, E, F) but not in the ice. The distribution of DMSO in the unfrozen solution outside the cell is non-uniform with the relative concentration at the center of the extracellular gap being greater than that near the ice interface (Figure 2E).

Inside the cell, there are several signals of interest. The Amide I intensity inside the cell is non-homogeneous (Figures 2C, G) and reflects the presence of subcellular structures. The nucleus is one example of a subcellular structure. A characteristic peak for DNA (784 cm^−1^) can be identified inside the cell (Figure 2D) and overlaps with regions of high Amide I concentration.

For the purpose of this investigation, we are interested in the distribution of DMSO inside the cell. Signal for DMSO is present both in the cytosol and the nucleus of cell with the ratio of DMSO in the nucleus 95% of that observed in the cytosol for the cell shown in Figure 2. The DMSO heat map and line scans (Figures 1C, F) demonstrate that there are non-uniformities of DMSO distribution in the cell. Along the cell membrane, there is also a change in DMSO intensity when traveling across the membrane (Figure 1L). Depending on the location where the line scan is taken around the periphery of the cells, the DMSO intensity will also vary inside of the cell as well. As a result, the partitioning ratio is calculated based on area averages for intracellular and extracellular spaces. The quantitative value of signal intensity varied from experiment to experiment and as a result, it is the relative concentration of a given substance that is compared for each image.

A method of quantifying the heterogeneity of a given signal is to calculate the coefficient of variation for that signal. The coefficient of variation in DMSO is calculated for all of the cooling rates, DMSO concentrations, and cell types studied in this investigation (Table 2). There is in general little variation in the coefficient of variation calculated over the different experimental conditions studied. The exception is the coefficient of variation for DMSO seen in Jurkat cells frozen at 1°C/min and 1% DMSO concentration (0.42).

**TABLE 2:** Coefficient of variation of the relative concentration of the Raman signal for DMSO inside of the cellular space for each cell type and condition.

Cell type	DMSO concentration (% vol/vol)	Cooling rate (C/min)	Intracellular DMSO coefficient of variation	Standard error	N
Jurkat^a^	1	1	0.42	0.05	6
Jurkat	5	1	0.18	0.02	10
Jurkat	10	1	0.15	0.02	6
Jurkat	10	0.5	0.14	0.02	7
Jurkat	10	5	0.16	0.04	6
SN	10	1	0.17	0.02	6
hiPSCs	10	1	0.22	0.03	7

^a^ Indicates a statistical significance from the Jurkat Cells cooled at 1°C/min with a concentration of 1% DMSO of *p* < 0.05 between other cell types conditions described in this table.

### 3.2 Partitioning ratio varied with DMSO concentration and cooling rate

**FIGURE 3:**
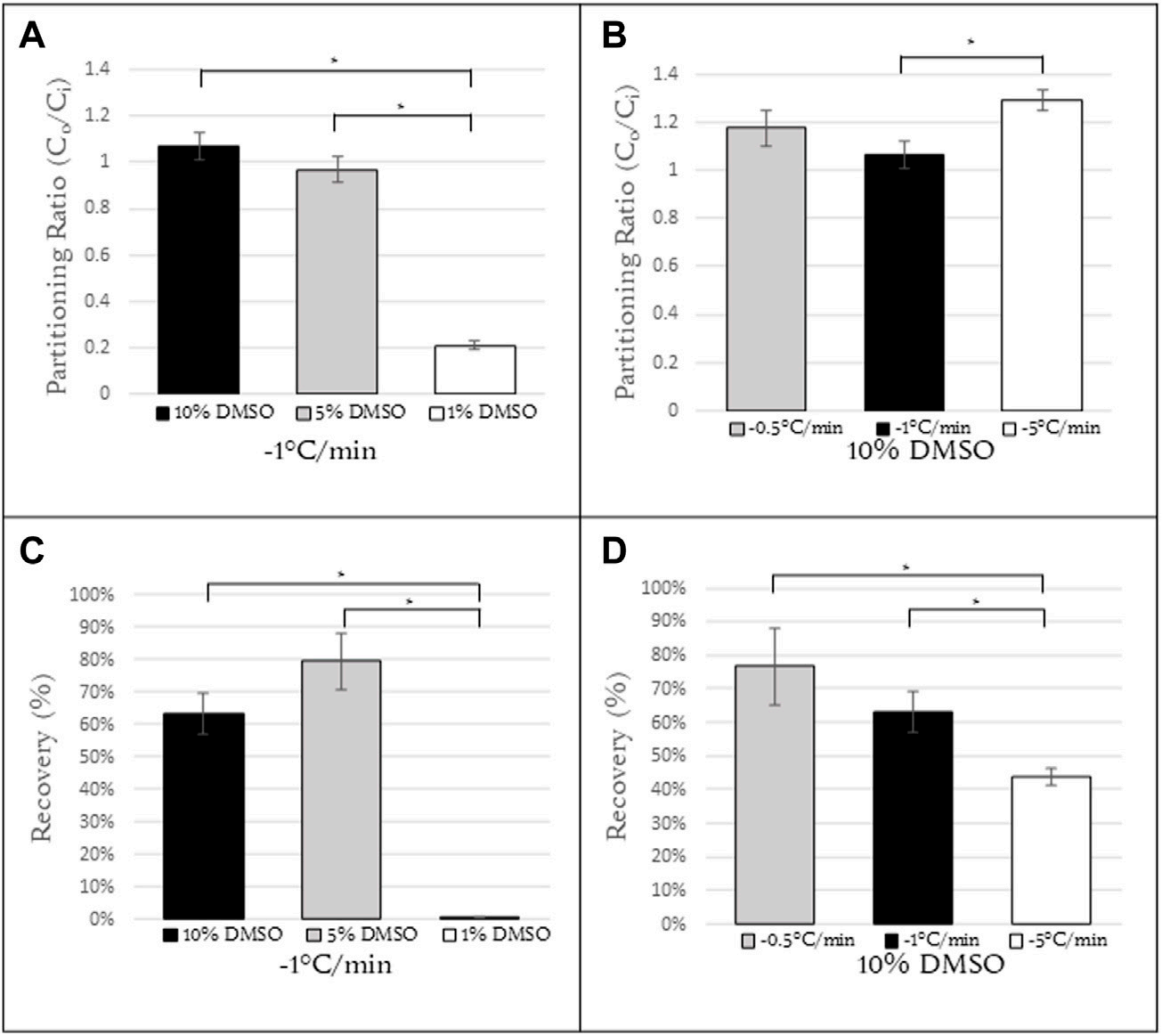
Partitioning ratios of Jurkat cells at **(A)** constant cooling rate of 1°C/min with concentrations of 10%, 5%, 1% DMSO (N = 6, N = 10, N = 6, respectively) and **(B)** constant concentration of 10% DMSO with cooling rates of 0.5, 1, and 5°C/min (N = 7, N = 6, N = 6, respectively). **(C)** Recovery calculations for Jurkat cells with the same conditions as **(A)**. **(D)** Recovery calculations for Jurkat cells with the same conditions as **(B)**. N = 9 for all samples in **(C,D)**.*Indicates a *p*-value < 0.05.

The cooling rate and the composition of the cryopreservation media are two factors that play an important role in cell survival. In order to characterize the influence of these two parameters on partitioning of DMSO, Jurkat cells were resuspended in DMSO-containing solutions (1, 5, 10% vol/vol) and cooled at a specified cooling rate (0.5, 1, 5, 10°C/min). The cells were held at −50°C, imaged, and the partitioning ratio of the cells quantified (Figures 3A, B). These same conditions were then used for freezing Jurkat cells and quantifying post-thaw recovery for the different freezing conditions (Figures 3C, D).

For a cooling rate of 1°C/min, the partitioning ratios for 10% and 5% DMSO were roughly 1 (1.07 ± 0.06 and 0.97 ± 0.05, respectively) and with the difference between them not statistically significant. However for a DMSO concentration of 1%, the partitioning ratio decreased to 0.21 ± 0.02, a statistically significant difference from that for both 10% and 5% DMSO (Figure 3A) (*p* < 0.05). When Jurkat cells were frozen using these same experimental conditions (1°C/min cooling rate and DMSO concentration) and post-thaw recovery determined, the post-thaw recovery of cells cryopreserved in 1% DMSO was considerably lower (*p* < 0.05) than that observed at 5% and 10% DMSO (Figure 3C).

For a 10% DMSO concentration, the partitioning ratio was calculated as a function of cooling rate. There was no statistically significant difference in the partitioning ratio observed at 1°C/min and 0.5°C/min (1.07 ± 0.06 and 1.18 ± 0.08, respectively). At a cooling rate of 5°C/min (1.30 ± 0.04), there was a statistically significant difference in partitioning ratio between this cooling rate and 1°C/min (Figure 3B). The partitioning ratio for cooling rates above 5°C/min could not be determined as the cells at those cooling rates exhibited high rates of intracellular ice formation. This issue will be discussed further below. Post-thaw recovery decreased with an increasing cooling rate (Figure 3D).

### 3.3 Cell type influenced partitioning ratio

**FIGURE 4:**
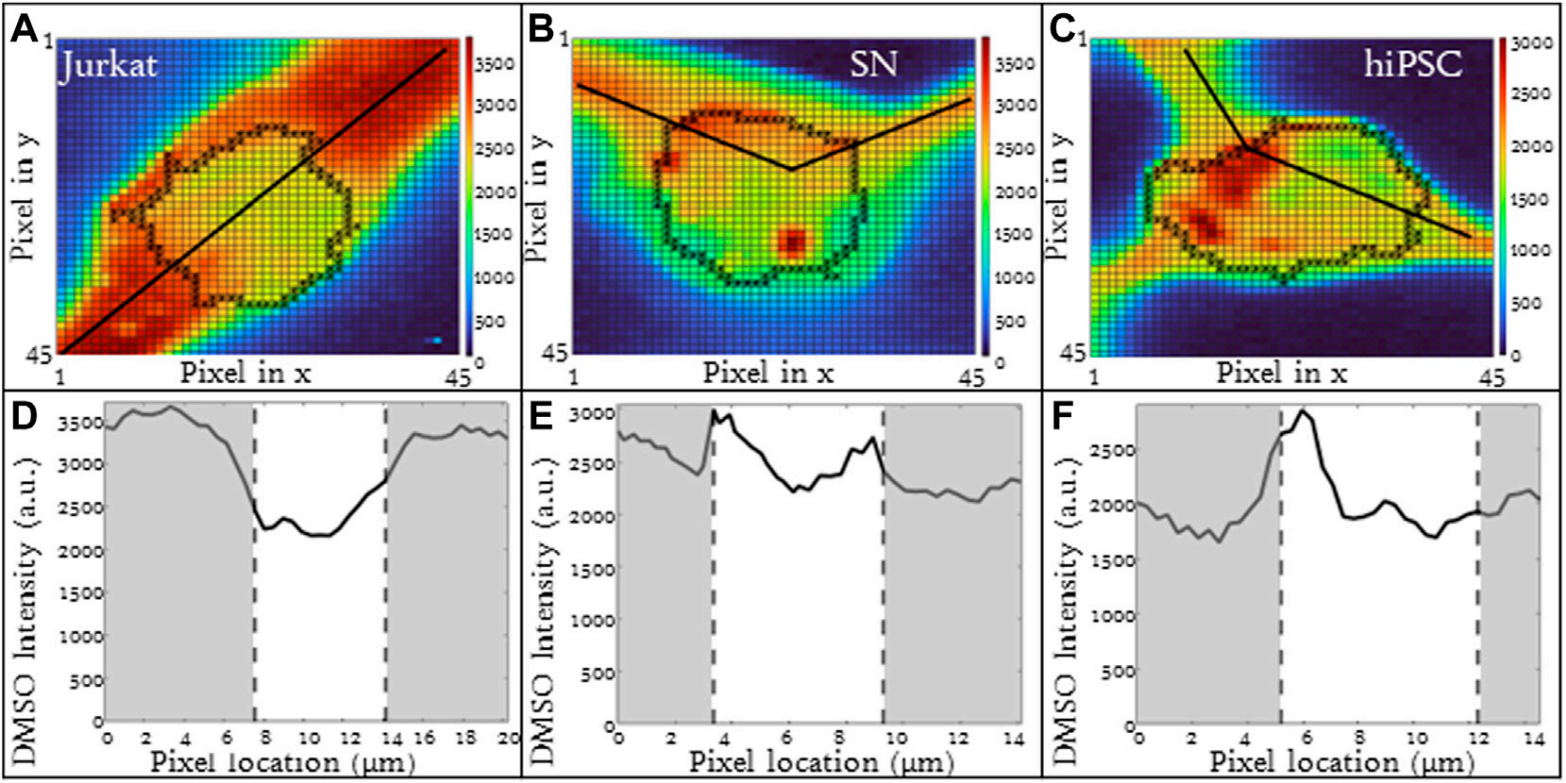
Raman heat maps of DMSO for a **(A)** Jurkat cell, **(B)** SN, and **(C)** hiPSCs in 10% DMSO frozen using a -1°C/min cooling rate and imaged at a temperature of −50°C. Heat maps show the intensity of DMSO. Line scans across the extracellular and intracellular space of each show DMSO variation for each cell type **(D)** Jurkat cell, **(E)** SN, and **(F)** hiPSCs. Gray areas indicate extracellular regions and white areas indicate intracellular regions. Pixels in **(A–C)** have a height and width of 333 nm.

Different cell types have different membrane characteristics and therefore may have different partitioning ratios. The influence of cell type on partitioning ratio was determined for three different cell types: Jurkat (model T-cell) cells, neuronal (SN) cells, and induced pluripotent stem cells (hiPSCs) in suspension. To observe differences in DMSO transport, all three cell types were cooled at the same rate of 1°C/min and CPA concentration 10%(vol/vol) DMSO. The partitioning ratio of SN and hiPSCs were less than that measured for Jurkat cells and the differences were statistically significant (Table 3). The heat maps for each cell (Figures 4A–C), demonstrates non-uniformity in the distribution of DMSO across the cell.

**TABLE 3:** Partitioning ratios of different cell types and standard error.

Cell type	Partitioning ratio (*C*_*o*_/*C*_*i*_)	Standard error	N
Jurkat	1.06	0.06	6
SN^a^	0.86	0.06	6
hiPSCs^a^ ^,^ ^b^	0.51	0.06	7

^a^ Indicates a two-sample t-test *p*-value <0.05 compared to Jurkat cells.

^b^ Indicates a Student’s t-test *p*-value <0.05 compared to SN cells.

The line scans for the three different cell types illustrate implications for the different partitioning ratios. For all three cell types studied, the relative DMSO concentration near the cell membrane is different than that observed in the center of the cell. For Jurkat cells, the relative DMSO concentration in the unfrozen solution outside the cell is high and the concentration decreases with proximity to the cell membrane. The DMSO concentration continues to decrease as you enter the cell and pass through the center of the cell. This concentration profile is consistent with that expected for a partitioning ratio > 1 (Figure 4D). In contrast, the relative extracellular DMSO concentration for SN decreased with proximity to the cell membrane with a rapid increase in DMSO concentration at the cell membrane and a graduate diminution of intracellular DMSO concentration at the center of the cell. This type of profile is consistent with a partitioning ratio < 1 (Figure 4E). Both the heat map and the line scan for DMSO distribution in hiPSCs differed from either SN or Jurkat cells (Figures 4C,F). There was a significant gradient in DMSO concentration across the cell and the overall partitioning ratio was lower.

### 3.4 Intracellular ice formation alters solute partitioning

**FIGURE 5:**
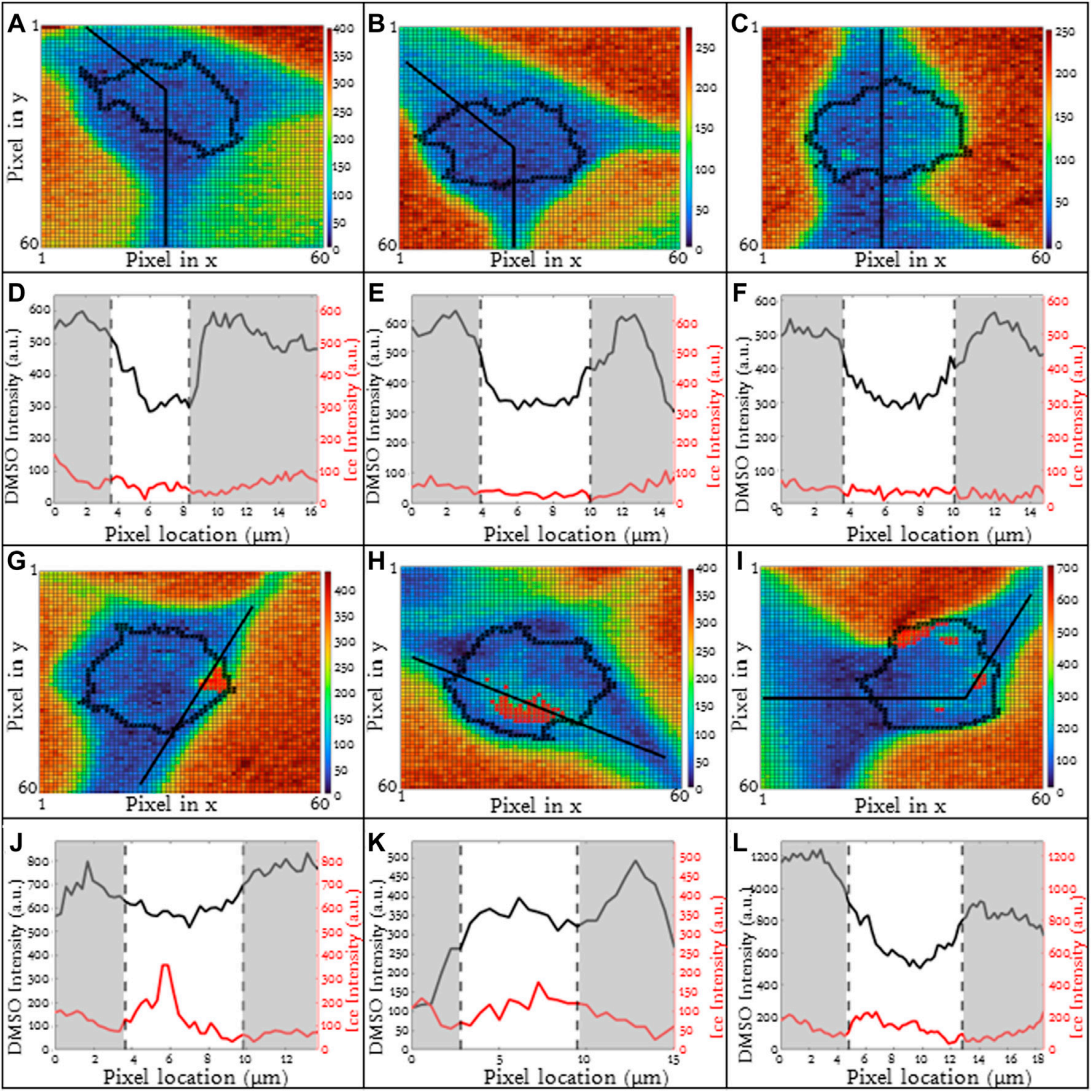
**(A–C)** Raman heat maps of three different Jurkat cells in 5% DMSO frozen using a −10°C/min cooling rate with no intracellular ice formation using the ice (OH) signal. The respective **(D–F)** line scans of DMSO and ice intensities across extracellular and cellular regions. **(G–I)** Raman heat maps of three different Jurkat cells in 5% DMSO frozen using a −10°C/min cooling rate with extensive intracellular ice formation using the ice (OH) signal. The respective **(J–L)** line scans of DMSO and ice intensities across extracellular and cellular regions. Pixels in **(A–C)** and **(G–I)** have a height and width of 250 nm. Red indicates intracellular ice formation and black represents the cellular boundaries. Gray areas indicate extracellular regions and white areas indicate intracellular regions in **(D–F)** and **(J–L)**.

For higher cooling rates, ice inside the cell is commonly observed (Yu et al., 2017). The formation of ice inside the cell may influence the concentration and redistribution of solute inside the cells (liquid water is removed from the cytosol to form ice). Jurkats frozen in 10% DMSO with a 10°C/min cooling rate included cells which exhibited intracellular ice formation and others which did not. The heat maps for three exemplary cells without ice are given (Figures 5A–C) and corresponding line scans are given as well (Figures 5D–F). The line scans for these three cells (Figures 5D, E) are fairly similar with a high relative concentration of DMSO in the unfrozen solution outside of the cell. The concentration of DMSO is lower inside the cell and the partitioning ratios for these three cells are 1.25, 1.07, and 1.34 respectively with heterogeneity being 0.16, 0.15, and 0.19 respectively.

In contrast, for the same cell type, CPA concentration and cooling rate, the presence of intracellular ice alters the distribution of DMSO across the cell. For three exemplary cells in which intracellular ice was observed, there was much greater variation in partitioning ratio (1.17, 0.46 and 1.22 respectively) (Figures 5G–I). The ice present in the cells is indicated in red (Figures 5G–I). Some of the variability in partitioning ratio may result from the varying amounts of ice present in the cell. The area fraction of the cell containing ice, AIC, is defined as the cross-sectional area of ice to the cross-sectional area of the cell. For the three cells containing intracellular ice in Figure 5, the AIC was calculated to be 0.07, 0.09, and 0.10 (Figures 5G–I). The line scans for the cells (Figures 5J–L) also demonstrated variation in DMSO concentration with the heterogeneity for the three different cells as 0.17, 0.25, and 0.22 respectively.

## 4 Discussion

### 4.1 Spatial distribution of DMSO

The outcome of this investigation found that ice is present outside the cell and that the signal for ice is high and uniform. The distribution of DMSO in the unfrozen solution outside of the cell is more complex. Specifically, the relative DMSO concentration in the center of the gap between adjacent ice crystals is greater than that observed adjacent to the ice interface. This observation is consistent with a study by Yu and colleagues (Yu et al., 2019) in which the concentration of sucrose in the gap between adjacent ice crystals was higher at the center when compared to the ice interface (Figure 2F). This outcome implies that there is some solute polarization taking place during freezing. One potential explanation why the relative concentration of DMSO is lower at the interface of the ice is that liquid water is still able to diffuse to the ice interface.

The interior of the cell is complex and within the spatial resolution of the system (300 nm), DMSO has penetrated everywhere in the cell. DMSO is observed inside the nucleus. This outcome is consistent with the study of Finan and Guilak (2010) who observed transport across the nuclear membrane for water. Differences in the ability of DMSO to penetrate different subcellular structures (mitochondria, cytoskeleton, etc) could explain the heterogeneity of DMSO concentration across the cell. While different cell types show different partitioning ratios, coefficient of variation as a measure of heterogeneity in the distribution of DMSO is consistent across cell types tested. The exception in this situation is the condition of Jurkat cells that were frozen in 1% DMSO at 1°C/min. This particular condition will be discussed below in more detail.

The relative intracellular distribution of DMSO exhibits variation across the cell with relative DMSO concentration at the cell membrane being higher than the cytoplasmic region. This is seen with the boundary layer that DMSO forms around the cell with a change in DMSO intensity traveling across the cell membrane. This is consistent with the work of Yu and colleagues (2017) where a change in DMSO intensity occurs across the cell membrane and unfrozen solution. This gradual change in DMSO across cellular space is consistent with Yang et al. (2019) observation that DMSO has a much slower cellular penetration compared to water.

### 4.2 Influence of DMSO concentration and cooling rate on partitioning and post thaw recovery for jurkat cells

In this investigation, high rates of post-thaw recovery are observed for the cells cryopreserved in 5 or 10% vol/vol DMSO and using a 1°C/min cooling rate. This outcome is consistent with other groups (see Li and colleagues 2019 for review). For this cell type and freezing conditions, both of these DMSO concentrations demonstrated similar partitioning ratios (0.97 and 1.07). At −50°C, the average concentration of DMSO in extracellular channels is nearly equal to the average concentration of DMSO in the intracellular space.

When concentration of DMSO is low (1% (vol/vol)) for the same cooling rate, the partitioning ratio decreased significantly and not surprisingly the post-thaw recovery was also very low. It is noteworthy that for this concentration of DMSO, the partitioning ratio measured suggested that the concentration of DMSO inside the cell was greater than the extracellular concentration of DMSO. This outcome would be expected if the permeability of DMSO was much lower at lower concentrations trapping DMSO inside the cell during cooling. Studies by a variety of investigators have shown that increasing concentrations of DMSO correlate with increasing permeability of the cell to DMSO, water, ions, DNA, and certain drugs (Gurtovenko and Anwar, 2007; He et al., 2012). Other investigators have demonstrated that changes in membrane characteristics such as permeability and fluidity correlate with freezing outcome (Giraud et al., 2000; Blesbois et al., 2005). Specifically, reduced membrane permeability and fluidity result in poor freezing outcome. It is also noteworthy that the coefficient of variation was much higher for Jurkat cells frozen at 1% DMSO. It is also possible that the ability of DMSO to permeate different cellular structures is also diminished at the lower DMSO concentration, thereby trapping DMSO in certain regions.

For slower cooling rates (0.5°C and 1°C/min), there is sufficient time for the relative concentration of DMSO to equilibrate in the cell and in the extracellular channels. For Jurkat cells frozen at higher cooling rates, there was an increase in partitioning ratios. This suggests that the concentration of DMSO outside the cell is greater than that inside. This outcome is consistent with models of water and DMSO transport during freezing (Kleinhans, 1998) in that permeability of the cell membrane decreases with decreasing temperature, thereby reducing the efflux of water and DMSO outside the cell. It is noteworthy that (Akhoondi et al., 2012) observed permeation of water and DMSO at low temperatures using Fourier Transform Infrared (FTIR) spectroscopy. The outcome of this investigation suggests that permeation of water and DMSO does decrease with decreasing temperature but transport of water, in particular, can still take place at the low temperatures used in this investigation allowing for different partitioning ratios to be observed with different cooling rates even with the same relative concentration. There was not a significant increase in the partitioning ratio from 0.5°C/min Jurkat cells to 1°C/min. However, there was a significant increase in the partitioning ratio from 1°C/min to 5°C/min suggesting that the cooling rate is too fast for cells to fully dehydrate. For Jurkat cells, there is a range of acceptable partitioning ratios and corresponding freezing protocols for Jurkat cells. As the cooling rate increases, the relative concentration of DMSO is higher in the extracellular space than the intracellular space. This data suggests that a partitioning ratio closer to 1 correlates to higher levels of post-thaw recovery. The recovery observed at higher and lower partitioning ratios (1.30 and 0.21, respectively) was lower.

As the cooling rate increases, the fraction of cells exhibiting intracellular ice increase (Toner and Cravalho, 1990) and this has been observed in a wide variety of cell types. The presence of ice inside the cell changes the partitioning and the distribution of DMSO across the cell (Figure 5). The presence of intracellular ice increases significantly the heterogeneity of DMSO distribution across the cell. This outcome is consistent with a study by Muldrew and McGann (1994) in which they hypothesized that the mechanism of damage associated with intracellular ice formation is associated with osmotic stresses resulting from the formation of ice and the rejection of solute (DMSO) from the ice phase.

### 4.3 Different cell types have different partitioning properties

As shown above, different cell types exhibited different partitioning ratios. However, the heterogeneity across the cell was consistent for the different cell types (Table 2). The differences in partitioning ratio must be examined in the context of both the cell type as well as other factors such as freezing conditions. For example, Li and colleagues (2021) observed an optimum cooling rate of −3°C/min for SN. The cooling rate used for the SN studies in this investigation was −1°C/min. It is possible that increasing the cooling rate to −3°C/min may have resulted in an increase in the partitioning ratio closer to 1 as well as a higher post thaw recovery. For hiPSCs, the configuration of the cells is important. The studies in this investigation involved single hiPSCs. When attached to other hiPSCs, significant changes in freezing response were observed (Li et al., 2018; Li et al., 2019; Li et al., 2020; Li et al., 2021). It is possible that attachment to other cells changes the membrane permeability, the partitioning of DMSO and therefore the freezing response.

As described above, DMSO and cooling rate can influence cell permeability. It is unclear whether this relationship is the same for the sensory neurons and induced pluripotent stem cells as it was for the Jurkat cells tested with different conditions. Further work will be needed to quantify these effects and therefore know if different cooling rates or compositions are better for SNs and hiPSCs.

## 5 Conclusion

This study leverages Raman microspectroscopy to define partitioning ratio of a common cryoprotectant DMSO as the spectral intensity of DMSO in the extracellular space to its spectral intensity in the cellular space. These calculations were performed by identifying key areas of the image such as the cell, the extracellular unfrozen section in between ice crystals, and the ice crystals themselves. These areas were determined using derivative thresholding and peak association and used to calculate partitioning ratio and heterogeneity of cryoprotectants inside a cell. The partitioning ratio gave insights to the concentration of DMSO in the extracellular spaces and inside of the cell for different cooling rates, DMSO concentrations, and cell types. For cells and freezing conditions that show promising recovery rates, the partitioning ratio is close to 1. The outcome of this investigation has demonstrated that concentration, cooling rate and cell type changes the partitioning ratio. Partitioning ratios close to one correlate with higher post-thaw viability and both lower and higher partitioning ratios correlate with poorer post-thaw viability. Differences in partitioning ratios between different cell types may suggest that different freezing conditions are needed for different cell types, which has been the conventional wisdom for many years. Further work is needed to quantify the relationship amongst membrane properties (fluidity and permeability), partitioning, cooling rate, and post-thaw survival and compare DMSO to other common cryoprotectants. Low-temperature Raman spectroscopy is a powerful tool that can be used to characterize freezing response and can help us gain insight, in particular, into cells with poor freezing response.

## Data Availability

The raw data supporting the conclusions of this article will be made available by the authors, without undue reservation.
